# The South African Society of Psychiatrists/Psychiatry Management Group management guidelines for adult attention-deficit/hyperactivity disorder

**DOI:** 10.4102/sajpsychiatry.v23i0.1060

**Published:** 2017-04-25

**Authors:** Renata Schoeman, Rykie Liebenberg

**Affiliations:** 1 Stellenbosch University Business School, Stellenbosch University, South Africa; 2 Private Practice, South Africa

## Abstract

**Disclaimer:**

These guidelines do not aim to provide a comprehensive review of all the pertinent literature comprising the evidence base and, as such, should be utilised in conjunction with other guidelines as well as the responsibility of practitioners to maintain a high level of personal knowledge and expertise. Despite the known efficacy of treatment and the substantial costs of untreated attention-deficit/hyperactivity disorder (ADHD), access to healthcare and treatment is not a given for many patients in South Africa (SA). In SA, there is poor identification and treatment of common mental disorders at primary healthcare level and limited access to specialist resources with a service delivery and treatment gap of up to 75%. Medication options are also often limited in emerging markets and in SA psychiatrists, and patients do not have access to the medication armamentarium available in established markets. Furthermore, the majority of South Africans currently utilise the public healthcare sector and may not have access to treatment options referred to in these guidelines. These guidelines should therefore not be seen as a policy document.

**The process:**

The South African Society of Psychiatrists’ Special Interest Group (SIG) for adult ADHD was launched on 25 September 2015, with doctors Rykie Liebenberg and Renata Schoeman as convenor and co-convenor, respectively. The overall objective of the ADHD SIG is to improve the basket of care available to patients with ADHD. This is only possible through a combined and concerted effort of individuals with a special interest in and passion for ADHD to improve knowledge about and funding for the care of individuals with the disorder. One of the specific aims of the ADHD SIG was to develop South African guidelines for the diagnosis and treatment of adult ADHD specifically and update guidelines for the treatment of child, adolescent and adult ADHD. Dr Schoeman has recently completed her MBA at the University of Stellenbosch Business School with a thesis entitled ‘A funding model proposal for private health insurance for adult attention-deficit/hyperactivity disorder in the South African context’. This is first South African study exploring the situation with regard to the prevalence and treatment of adult ADHD. Dr Schoeman was tasked by the SIG with the drafting of guidelines. Dr Liebenberg provided valuable input. The guidelines were then circulated to the SIG members, as well as the Chair of the Public Sector SIG, for written feedback and evidence-based suggestions which were then incorporated into the guidelines. The final guidelines were circulated for written approval by the SIG members, followed by formal approval at a SIG meeting held on 14 August 2016, after which it was submitted to the South African Society of Psychiatrists (SASOP) and Psychiatry Management Group (PsychMG) boards for recommendation and ratification.

## Introduction

‘Mental restlessness’ was first described by Sir Alexander Crichton in 1798,^1^ while ‘Fidgety Philip’ (a popular storybook character and now also an allegory for children with attention-deficit/hyperactivity disorder [ADHD]) was created by Heinrich Hoffmann in 1844.^2^ Sir George Still’s Goulstonian Lectures,^3^ describing children with restlessness, inattention and impulsiveness, can be considered the starting point of the description ‘attention-deficit/hyperactivity disorder’ (ADHD) as we know it.

The notion that ADHD is a disorder of childhood prevailed until the 1990s. Rigorous research, including longitudinal studies, and public awareness highlighted the presence of ongoing symptoms in 65% of adult patients.^4,5^ Adult ADHD is now a recognised problem. Associated symptoms of ADHD include behavioural, cognitive, emotional and social problems. Problems with planning, task initiation, task completion, impatience and impulsivity can cause numerous work-related and interpersonal problems.

## Prevalence

ADHD is the most common psychiatric disorder in children – affecting 2.0% – 16.0% of the school-age population.^6^ The population prevalence for ADHD is estimated as 3.0% – 5.0%.^7^ It is now widely accepted that an estimated 60.0% – 70.0% of patients’ symptoms persist into adulthood,^8^ with estimates of the prevalence of adult ADHD between 2.5% – 4.3%.^9,10,11^ ADHD is more frequent in men than in women in the general population, with a ratio of approximately 1.6:1 in adults. Women are more likely than men to present primarily with inattentive features. The increased diagnosis of ADHD over the past decade seems to reflect improved criteria for the identification of ADHD in adults and female patients.^12,13,14,15,16^

The specific lifetime prevalence of ADHD in South Africa (SA) is unknown. The South African Stress and Health study, a nationally representative household survey of 4351 adults, conducted between 2002 and 2004, investigated the prevalence and treatment access and use for mental health disorders. Unfortunately, results were grouped as anxiety disorders, mood disorders, substance use disorders (SUD), and ‘any other disorder’. ADHD would be included within the last group, with a prevalence of 30.3%.^17,18^

In the first South African study exploring the situation with regard to the prevalence and treatment of adult ADHD,^19^ extrapolating the known prevalence information to the South African context, the expected number of adults between the age of 20 and 50 years affected by ADHD was calculated to be between 771 264 (3%) and 1 285 439 (5%). In this triangulated study (consisting of a database analysis of one of the largest medical scheme administrators in SA in terms of medical, pharmaceutical and claims data for the treatment of ADHD over a 2-year period; a survey with psychiatrists in private practice who manage adult ADHD; and a qualitative analysis of in-depth interviews with key opinion leaders on their experiences in working with adults with ADHD), the population prevalence of adult ADHD was estimated at 1.09%, whereas the prevalence in clinical psychiatric settings was as high as 52.5%, of which 13.68% were patients with newly-diagnosed ADHD. Conditions comorbid with adult ADHD are common with psychiatric conditions in up to 20.43% of individuals. In this study, lack of knowledge of adult ADHD and lack of funding for the treatment thereof were identified as the two main barriers to diagnosis and treatment. It is therefore possible that these prevalence rates are underestimating the true prevalence of adult ADHD in SA.

## Costs

ADHD is a costly, chronic disorder, with significant impact on the quality of life of patients and their families. The burden of disease is significant, with the disability-adjusted life years (DALYs) calculated as 424 per 100 000.^20^ The economic burden of adult ADHD ($3020 per patient per annum) is less than those of depression and diabetes, but more so than seasonal allergy.^21^

Significant comorbidity, estimated at more than 50% with ADHD, contributes to the burden of disease and reduced quality of life of patients with ADHD. Adults with ADHD are more likely to suffer from comorbid medical disorders (e.g. asthma, unplanned pregnancies and sexually transmitted diseases), as well as psychiatric disorders (e.g. mood disorders, anxiety disorder, substance abuse disorders and behavioural disorders).^22,23^

(Untreated) ADHD also impairs educational attainment and employment status, impairs work performance (reduced productivity and increased absenteeism), adversely affects interpersonal relationships (irritability and low frustration tolerance) and adds to significant societal costs (because of substance abuse and accidents).^24,25,26,27,28,29,30,31^

Persons with ADHD have lower self-esteem and general happiness with life, higher divorce rates and problems keeping friends and are at risk of forensic contact.^26,32,33,34,35^

In a South African study,^19^ the presence of adult ADHD more than doubled the healthcare costs of medical scheme beneficiaries. However, the costs attributable to direct costs of ADHD formed only 0.56% of the total value of claims. Of this, medication was the largest expense (5.47% of total costs and 38.48% of direct costs), whereas psychiatric services formed only 3.27% of the direct costs. This is in agreement with international findings^28^ where the cost of medication for treatment of ADHD amounted to 7% of all direct and indirect costs. This would suggest that pharmacological treatment of ADHD is cost-effective.

Individuals with untreated ADHD, their families and other caregivers must be made aware of the impact this disorder may have on them at every stage of life and, correspondingly, the improved outcomes that can be achieved with the successful management of ADHD.

## Diagnosis and clinical characteristics

The core triad of ADHD is a persistent pattern of inattention or hyperactivity–impulsivity that interferes with functioning. This is accompanied by associated behavioural, cognitive, emotional and social problems which can lead to work-related and interpersonal difficulties (see Box 1 for *The Diagnostic and Statistical Manual of Mental Disorders* (fifth edition) (DSM-5) criteria for ADHD).^36^

BOX 1 DSM-5 criteria for ADHD.

- A persistent pattern of inattention and hyperactivity-impulsivity that interferes with functioning or development, as characterised by (1) and (2):
  1. **Inattention:** Six (or more) of the following symptoms have persisted for at least 6 months to a degree that is inconsistent with developmental levels and negatively impacts directly on social and academic or occupational activities:
    Note: The symptoms are not solely a manifestation of oppositional behaviour, defiance, hostility or failure to understand tasks or instructions. For older adolescents and adults (age 17 years and older), at least five symptoms are required:
    - Often fails to give close attention to details or makes careless mistakes in schoolwork, at work or during other activities (e.g. overlooks or misses details, work is inaccurate).
    - Often has difficulty sustaining attention in tasks or play activities (e.g. has difficulty remaining focused during lectures, conversations or lengthy reading).
    - Often does not seem to listen when spoken to directly (e.g. mind seems elsewhere, even in the absence of any obvious distraction).
    - Often does not follow through on instructions and fails to finish schoolwork, chores or duties in the workplace (e.g. starts tasks but quickly loses focus and easily side tracked).
    - Often has difficulty organising tasks and activities (e.g. difficulty managing sequential tasks; difficulty keeping materials and belongings in order; messy, disorganised work; has poor time management; fails to meet deadlines).
    - Often avoids, dislikes or is reluctant to engage in tasks that require sustained mental effort (e.g. schoolwork or homework; for older adolescents and adults, preparing reports, completing forms and reviewing lengthy papers).
    - Often loses things necessary for tasks or activities (e.g. school materials, pencils, books, tools, wallets, keys, paperwork, eyeglasses and mobile telephones).
    - Is often easily distracted by extraneous stimuli (for older adolescents and adults, may include unrelated thoughts).
    - Is often forgetful in daily activities (e.g. doing chores, running errands; for older adolescents and adults, returning calls, paying bills and keeping appointments).
  2. **Hyperactivity and impulsivity:** Six (or more) of the following symptoms have persisted for at least 6 months to a degree that is inconsistent with developmental levels and negatively impacts directly on social and academic or occupational activities:
    Note: The symptoms are not solely a manifestation of oppositional behaviour, defiance, hostility or a failure to understand tasks or instructions. For older adolescents and adults (age 17 years and older), at least five symptoms are required.
    - Often fidgets with or taps hands or feet or squirms in seat.
    - Often leaves seat in situations when remaining seated is expected (e.g. leaves his or her place in the classroom, in the office or other workplace or in other situations that require remaining in place).
    - Often runs about or climbs in situations where it is inappropriate. (Note: In adolescents or adults it may be limited to feeling restless.)
    - Often unable to play or engage in leisure activities quietly.
    - Is often ‘on the go’, acting as if ‘driven by a motor’ (e.g. is unable to be or uncomfortable being still for extended time, as in restaurants or meetings; may be experienced by others as being restless or difficult to keep up with).
    - Often talks excessively.
    - Often blurts out an answer before a question has been completed (e.g. completes people’s sentences; cannot wait for his or her turn in conversation).
    - Often has difficulty waiting his or her turn (e.g. while waiting in line).
    - Often interrupts or intrudes on others (e.g. butts into conversations, games or activities; may start using other people’s things without asking or receiving permission; for adolescents and adults, may intrude into or take over what others are doing).
- Several inattentive or hyperactive-impulsive symptoms were present prior to 12 years of age.
- Several inattentive or hyperactive-impulsive symptoms are present in two or more settings (e.g. at home, school or work, with friends or relatives, in other activities).
- There is clear evidence that the symptoms interfere with or reduce the quality of social, academic or occupational functioning.
- The symptoms do not occur exclusively during the course of schizophrenia or another psychotic disorders and are not better explained by another mental disorders (e.g. mood disorder, anxiety disorder, dissociative disorder, personality disorder, substance intoxication or withdrawal).

*Source*: American Psychiatric Association (APA)^36^

Inattention manifests behaviourally in ADHD as difficulty sustaining focus, wandering off tasks, lacking persistence, paralysing procrastination, poor time management, inefficiency and being disorganised.

Hyperactivity refers to excessive motor activity when it is not appropriate or excessive fidgeting, tapping or talkativeness. In adults, hyperactivity may manifest as extreme restlessness or wearing others out with their activity, being a workaholic, excessive talkativeness, being overscheduled and feeling overwhelmed.

Impulsivity refers to hasty actions that occur in the moment without forethought and could have a high potential for harm to the individual. This may reflect reward dependence and a need for immediate gratification. Impulsive behaviours may manifest as social intrusiveness, a low frustration tolerance, mood lability and losing one’s temper, making important decisions without consideration of long-term consequences (e.g. taking a job without adequate information, or impulsively quitting a job, ending relationships, or driving too fast) and addictive behaviours.

These core symptoms should be evident since childhood, with evidence of several symptoms being present since before the age of 12 years. Also, substantial symptoms causing significant impairment should be present in more than one setting (e.g. home, school and work). Some of the impairments related to adult ADHD include job failure or under-employment, complications such as drug dependence, driving accidents, unwanted pregnancies and sexually transmitted diseases, and even a life of perpetual failure.

Typically, symptoms vary depending on context within a given setting. Adult recall of childhood symptoms tends to be unreliable. Confirmation of the presence of symptoms and the impact thereof across various settings typically cannot be done accurately without consulting informants who have seen the individual in those settings. Obtaining collateral information is therefore beneficial – if not crucial.

Symptoms often appear to decrease over time – in number and in severity. However, adults are often more adept at managing these symptoms. Some adults compensate for ADHD-related impairment by choosing lifestyles and careers that suit them. Although they may appear to function well, high amounts of energy and time are used to accomplish tasks.

A field trial in a representative large sample (*N* = 4000) of 18–19-year-old adults^14,15^ indicated a 27% increase (from 2.8% to 3.55%) in the expected prevalence of ADHD when comparing DSM-IV to DSM-5 criteria. However, the study supported lowering the symptomatic threshold for diagnosing ADHD in adults, with the best symptomatic cut-off in the number of symptoms for predicting impairment being five symptoms of inattention and four symptoms of hyperactivity-impulsivity.

Based on these criteria, three types of ADHD are identified:

1. ADHD combined type: If both criteria 1A and 1B are met for the past 6 months
2. ADHD predominantly inattentive type: If criterion 1A is met, but criterion 1B is not met for the past 6 months
3. ADHD predominantly hyperactive-impulsive type: If criterion 1B is met, but criterion 1A is not met for the past 6 months.

Further distinctions are made with regard to severity:

- Mild: Few, if any, symptoms in excess of those required to make the diagnosis are present, and symptoms result in no more than minor impairments in social or occupational functioning.
- Moderate: Symptoms or functional impairment between ‘mild’ and ‘severe’ are present.
- Severe: Many symptoms in excess of those required to make the diagnosis, or several symptoms that are particularly severe are present, or the symptoms result in marked impairment in social or occupational functioning.

A course specifier for partial remission is specified where full criteria were previously met, fewer than the full criteria have been met for the past 6 months, and the symptoms still result in impairment in social, academic or occupational functioning. Furthermore, provision has been made for patients who present with symptoms characteristic to ADHD that cause clinically significant distress or impairment in social, occupational or other important areas of functioning predominate but do not meet the full criteria for ADHD. This is done by recording ‘other specified attention-deficit/hyperactivity disorder’ followed by the specific reason (‘with insufficient inattention symptoms’). If the clinician chooses not to specify the reason for partial fulfilment of criteria, or when there is insufficient information to make a more specific diagnosis, the diagnosis will be ‘unspecified attention-deficit/hyperactivity disorder’.

## Assessment

Recognition of adult ADHD as a chronic disorder which needs chronic treatment is crucial. ADHD is a clinical diagnosis, which should only be made by a specialist psychiatrist, paediatrician or other healthcare professional with training and expertise in the diagnosis of ADHD. Adults with suspected ADHD whom have previously been diagnosed with ADHD during childhood with symptoms suggestive to ongoing ADHD can consult general adult psychiatric service for confirmation of diagnosis and ongoing treatment. Adults with suspected ADHD without previous diagnosis during childhood should be assessed by a specialist psychiatrist with adequate training and experience in assessments of adults with suspected ADHD for confirmation of diagnosis and treatment initiation.^37^

It is important to consider the history of presenting complaints, but also to use (semi-)structured interviews, rating scales, school- or work-related assessments, social functioning assessments and collateral information in the diagnosis of the disorder.

### Screening

Commonly used rating scales for screening adult ADHD include the World Health Organisation Adult ADHD Self-Report Scale (ASRS) Symptom Checklist,^38^ the Barkley adult ADHD rating scale,^39^ the Brown ADD Scale Diagnostic Form (BADDS),^40^ the ADHD Rating Scale,^41^ the Conner’s Adult ADHD Rating Scale (CAARS) (DSM–IV),^42^ the Wender Utah Rating Scale (WURS),^43^ and the Wender-Reimherr Adult Attention Deficit Disorder Scale.^44^ The WHO ASRS is the best researched, and the shortened 6-item version has a sensitivity of 68.7% and specificity of 99.5% with total classification accuracy of 97.9% evaluated using population survey data. However, specificity of this and other screening tools may be lower within clinical samples with high rates of other mental health disorders, and positive screens should always be followed by full diagnostic evaluations based on clinical interview data.

### Clinical evaluation

The clinical interview is one of the cornerstones of the assessment process in diagnosing adult ADHD. Although various standardised, structured interviews and rating scales are available, these cannot replace the clinical assessment, but can add some rigor, standardisation and a quantifiable dimension to the areas being evaluated.

For the main diagnostic assessment, the use of the following structured diagnostic interviews may be considered: the Brown Adult ADHD Diagnostic Interview,^40^ the Conners Adults ADHD Diagnostic Interview for DSM-IV (CAADID),^45^ or the Diagnostic Interview for ADHD in adults (DIVA).^46^

A thorough interview is needed, documenting present symptoms and functionality across all spheres of the patient’s life. An assessment of the following would be essential: exploring the presence of the core symptoms of ADHD (inattention, hyperactivity and impulsivity), establishing the pervasiveness thereof throughout the individual’s lifespan and across different settings (work, school and social domains) and the presence of significant functional impairment throughout the lifespan and across different settings. It has to be noted that the expression of ADHD in adults differs to some extent from that in children, and the diagnostic descriptions of some of the features need to be adapted to adult expression of the disorder (physical over-activity in childhood could be replaced in adulthood by constant mental activity, feelings of restlessness and difficulty engaging in sedentary activities). The Barkley Functional Impairment Scale for adults^39^ and the Weiss Functional Impairment Rating Scales (WFIRS)^47^ are useful measures to assess the impact of symptoms on clinically relevant domains of functioning.

Differential diagnoses (e.g. a lack of effort, poor vocational match, or transient situational or environmental circumstances) and other psychiatric or medical diagnosis (and treatment thereof) that can explain the symptoms should be excluded. Furthermore, a thorough evaluation for the presence of comorbid psychiatric disorders (including substance use) is essential.

Comorbidity in individuals with adult ADHD is very common. Comorbid disorders can be the presenting problem in many adults and is a confounding factor in the diagnosis of ADHD, with a negative impact on treatment decisions and response. International studies indicated that the majority of adults with ADHD have complicating and clinically significant comorbid psychopathology, with more than one psychiatric disorder in up to 87%, whereas at least 57% of individuals have more than two psychiatric disorders.

In a South African database analysis,^19^ psychiatric comorbidity was also more prevalent in individuals with adult ADHD than in the general population for both anxiety disorders (13.1% vs. 8.1%) and mood disorders (13.8% vs. 4.5%). The presence of adult ADHD also more than tripled the prevalence of multiple comorbidity (9.09% vs. 3.9%). The most common psychiatric comorbidities were adjustment disorders in 29.5% of patients with comorbid psychiatric conditions (2.7% of the sample), followed by non-organic sleep disorders in 19.4% (1.8% of the sample) and anxiety disorders in 13.1% (1.2% of the sample). Mood disorders occurred as comorbid disorders in 13.8% of the patients (1.2% of the sample), whereas substance-related disorders occurred in 2.3% of adults with ADHD (0.2% of the sample).

In clinical psychiatric practice settings,^19^ psychiatrists indicated the presence of comorbid disorders in patients with adult ADHD as follows: anxiety disorders in 40.33% (the most prevalent being generalised anxiety disorder), SUD in 33.81% (with alcohol abuse being the most common substance of choice), major depressive disorder in 33.28% and bipolar mood disorder in 13.75%. Personality disorders as a group (mostly cluster B) were present in 28.26% of patients with adult ADHD.

Although a diagnosis based only on self-report is possible, such an approach may lead to an under- or over-diagnosis of ADHD, and it may be more reliable to obtain collateral information from other sources. Whenever possible, corroborating information should be obtained from a living parent, or older relative for childhood behaviour and a partner, relative or close friend for current behaviour and symptoms. This should include additional information on developmental and family history. Although Breda et al.^48^ found that collateral report has no incremental value in the evaluation of childhood ADHD symptoms in adults with a self-reported history of ADHD assessed in clinical settings, obtaining collateral information from parents on childhood ratings protect against possible student and adult malingering to obtain ADHD medications or accommodations.^49^

### Medical, laboratory and other special examinations

It is important that each patient should have a thorough medical evaluation which would include a complete medical history and comprehensive physical examination with the purpose of excluding causative or comorbid medical disorders, and a baseline safety assessment. The minimum requirements are the documentation of vital signs, weight and height. Screening for visual and auditory deficits or processing and integration problems should be considered and appropriate referrals to specialist optometrists and speech and language pathologist considered. Special examinations are a significant cost-driver in the management of ADHD and should not be requested routinely. ECGs should be requested in any patient with personal or familial cardiac risk factors. Liver function tests should be requested in patients with a history of liver disease prior to initiating treatment with atomoxetine. A comprehensive neurological examination should be considered if any symptoms suspicious of neurological disorders, including head injuries or symptoms of epilepsy, are present, with an appropriate referral to a neurologist or for an EEG where indicated. There are at present insufficient data for the use of brain imaging modalities in the diagnosis of adult ADHD. Although the US Food and Drug Administration (FDA) has approved an electroencephalogram-based instrument as a diagnostic aid, demonstrating higher resting theta:beta waves, for ADHD in children (as part of a complete medical and psychological evaluation), there is currently no evidence for the use thereof in the diagnosis of adults with ADHD.^50^ Brain mapping, event-related potentials and neuroimaging are also of limited clinical use. It lacks diagnostic specificity, is costly and is not readily available.

### Psychometric evaluation

There are currently no neuropsychological tests for ADHD with sufficient sensitivity and specificity to serve as an individual diagnostic test.^51,52,53^ Although specific neuropsychological tests, especially those examining executive dysfunction, can be useful, it should not be used in isolation as a diagnostic tool in the absence of a comprehensive clinical evaluation by an experienced clinician, but as complementary to diagnostic assessments in order to determine individual deficits and to suggest individualised interventions.^54^ Current neuropsychological tests based solely on executive function are of limited value, whereas multi-domain assessments and computerised assessments (the Quantified Behavioural Test QbTest),^55^ the MOXO d-CPT,^56^ and the Test of Variable of Attention (TOVA)^57^ have not yet established utility and cost-effectiveness.

Neuropsychological assessments should be considered when the functional impairment the patient is experiencing is more than what would be expected from the core symptoms alone. Neuropsychological testing is also useful to supplement core treatment in the development of personalised treatment approaches and interventions for educational or work purposes, as well as monitoring treatment response and identifying areas which may need further interventions, but should not be used to establish treatment response.^50^

Sensitive use of general intelligence tests can be useful to ascertain potential attainment and to diagnose co-morbid learning disabilities for educational interventions.^53^ Appropriate referral to an educational psychologist should be considered.

Interest, reward and educational achievement are important complicating factors during the assessment of ADHD. A significant percentage of those making suspect effort during neuropsychological evaluation will be diagnosed with ADHD using the most commonly employed assessment methods: interview alone (71%), interview and behaviour rating scales combined (65%) and an interview, behaviour rating scale and Continuous Performance Test (CPT) test combined (57%). It is therefore essential to evaluate task engagement and possible symptoms amplification during clinical evaluation.^58^

## Treatment

### Treatment goals

According to the WHO,^59^ mental health is ‘a state of well-being in which every individual realises his or her own potential, can cope with the normal stresses of life, can work productively and fruitfully, and is able to make a contribution to her or his community’. The initial goal of any intervention is therefore symptoms relief, followed by ongoing alleviation of symptoms accompanied by decreasing functional impairment and working towards optimal functioning. This will include the prevention and treatment of comorbid disorders, relapse prevention and the improvement of the quality of life of patients and their families.

### General aspects of treatment

As highlighted by the British Association for Psychopharmacology (BAP) guidelines,^50^ Maudsley guidelines^60^ and Schoeman,^19^ a comprehensive assessment and diagnostic certainty prior to initiating pharmacological treatment is crucial and treatment should be initiated at specialist level.

Good long-term clinical care is contingent on adequate patient education about the nature of the disorder and effects of treatment. Once the diagnosis has been confirmed, clinicians should provide sufficient information to empower patients to make informed decisions with regard to treatment options and designing interventions that meet the patient’s individual needs.

Pharmacotherapy remains the cornerstone of treatment. Contrary to the treatment of ADHD in children, pharmacological interventions are always first line in adults – based on the lack of efficacy of non-drug intervention in the absence of medical treatment.^60^ Drug treatment should be continued as long as clinically effective and should be reviewed at least annually. Effects of missed doses, planned dose reductions and periods of non-treatment should also be evaluated.^50^

Although pharmacotherapy plays a primary role in the treatment of ADHD, psychosocial interventions form an integral part of a comprehensive, multi-modal, management approach for adults with ADHD. Meta-analysis and systematic reviews have confirmed the superiority of combination (pharmacological and psychosocial) interventions. A multi-modal approach is also encapsulated in international guidelines (e.g. BAP and National Institute for Health and Care Excellence (NICE)) which recommend psychosocial treatments as complementary to psychopharmacological interventions to provide support, improve acceptance of diagnosis, and treat comorbidities and residual symptoms (which do not require additional psychopharmacological treatments).

### Cost-effectiveness of treatment

Few economic evaluation studies could be traced – all of them cost-effectiveness studies in children with ADHD. In a cost-effectiveness study, costs are related to a single common effect that may differ in magnitude between the alternative options or programmes^61^ (i.e. comparing treatment option A with treatment option B with regard to a certain outcome measure).

The first cost-effectiveness study in children with ADHD dates back to 2005.^62^ In this study, 579 children with ADHD were assigned to 14 months of medication management, behavioural treatment, both combined or community care. Services were tallied throughout the study, including medication, healthcare visits, behavioural treatments and rental costs. Provider specialty, total time and number of visits with providers were used to calculate costs, adjusted to FY 2000 dollars with the consumer price index. The authors found that treatment costs varied fourfold, with psychopharmacological management being the least expensive, followed by behavioural treatment, and then combined treatment. They concluded that medication only, although not as effective as combined treatment, is likely to be more cost-effective in routine treatment for children with ADHD.

Wu, Hodgkins, Ben-Hamadi, et al.^63^ did a systematic literature review of 13 economic evaluations of pharmacotherapies for children and adolescents with ADHD conducted between 1990 and 2011 in North America, Europe, Australia or New Zealand. There was consistent evidence that pharmacotherapies are cost-effective compared with no treatment or behavioural therapy. However, the data were not comprehensive enough to draw conclusions regarding the relative cost-effectiveness of different pharmacological agents.

### Pharmacological treatment

The first evidence for the effectiveness of stimulants in the treatment of ADHD dates back to 1937, when Bradley conducted a trial with Benzedrine in children with ADHD.^64^ The first double-blind placebo controlled trial in ADHD examining the efficacy of Dexedrine was done in 1967.^65^ Since then, many studies have been conducted and established the efficacy of both stimulant- and non-stimulant medications in the treatment of ADHD in children and adolescents, and more recently also in adults. Consistent with the catecholamine hypotheses of ADHD, the drugs that effectively treat the disorder are known to modulate catecholamine pathways.

Medications used in the treatment of ADHD include psychostimulants [e.g. methylphenidate (MPH) and amphetamines] and non-stimulants [e.g. atomoxetine, alpha_2_-adrenoceptor agonists (clonidine and guanfacine), tricyclic antidepressants (TCAs), bupropion, modafinil and venlafaxine]. Enhancement of dopaminergic and noradrenergic neurotransmission in the prefrontal cortex is probable critical to the therapeutic efficacy of ADHD medication^66^ (see Table 1 for the pharmacological classification of medication used for the treatment of ADHD).

**TABLE 1:** Pharmacological classification of medication used for the treatment of ADHD.

Neurotransmitter		Mechanism of action		
Noradrenaline selective	Noradrenaline and dopamine	Monoamine reuptake inhibitors	Monoamine releasing agents	Stimulant reuptake inhibitors
Atomoxetine	Methylphenidate d-amphetamine Lisdexamphetamine	Atomoxetine	d-amphetamine Lisdexamphetamine	Methylphenidate

*Source*: Dvorsky et al.^49^

Although pharmacotherapy plays a primary role in the treatment of ADHD, psychosocial interventions [psycho-education, cognitive behavioural therapy (CBT), supportive coaching or assistance with daily activities] are an integral part of management. Discussions on clinical efficacy are limited by the lack of head-to-head studies with adequate and unbiased methodology. In general, dopaminergic and noradrenergic agents can reduce the core symptomatology, though specific effects and side effects may vary between agents. Treatment choice would therefore also depend on factors such as patient preference and comorbid conditions, abuse potential, side effect profile and toxicity in overdose.

In terms of treatment, stimulants are by far the best studied (and most effective) treatment for ADHD across the lifespan.^67^ The three stimulants – MPH, pemoline (withdrawn from the market because of liver toxicity) and dextroamphetamine – have similar effects, with an average response rate of 70%.^8^

#### Methylphenidate (Ritalin^®^ IR & LA, Concerta^®^, HCL Douglas-methylphenidate^®^)

Various controlled studies have confirmed the effectiveness of MPH in about 57% of adults with ADHD.^68^ Meta-analyses of MPH in adults demonstrate similar drug response rates and effect sizes to those seen in children, although slightly lower. For direct comparison with placebo, effect sizes were calculated as 0.9 in favour of MPH.^69^ In a recent meta-regression analysis of 18 studies (2045 patients),^70^ MPH, at a mean dose of 57.4 mg/day, had a moderate effect on ADHD symptoms compared with placebo [standard mean difference (SMD) 0.57–0.58]. MPH improved ADHD symptoms in adults in a dose-dependent fashion, with an increase in efficacy (SMD 0.11–0.12) for every 10 mg increment of MPH. In another systematic review and indirect comparison meta-analysis of 22 placebo-controlled trials of 2203 adults with ADHD,^71^ conventional shorter-acting stimulants had a more favourable risk–benefit ratio than longer-acting stimulants and non-stimulants in adults. Short-acting stimulants also reach effect sizes of 4.32 versus an effect size of 1.35 for long-acting stimulants.

In addition to reducing the core symptoms of ADHD, stimulants improve associated features of ADHD, such as on-task behaviour, academic performance and social functioning. These effects appear to be dose-dependent and are evident across settings. In adults, emotional regulation, occupational problems and marital discord also tend to be reduced on treatment. Side effects (headache, reduced appetite, palpitations, nervousness, initial insomnia and dry mouth) are usually mild and transitory.^67^

The BAP guidelines^50^ and the NICE guidelines^37^ recommend stimulants, specifically MPH as the first-line treatment choice in adults. The European Network Adult ADHD (ENAA) consensus statement^67^ specifically recommends the use of extended release stimulants as treatment of choice for adults with ADHD. Long-lasting, extended-release formulations are preferred because of non-adherence, a lower abuse potential, fewer rebound symptoms and frequent dosing with short-acting formulations. The Maudsley guidelines recommend the use of modified-release MPH (because of the convenience of single-day dosage – therefore improving adherence) or multiple doses of immediate-release (greater flexibility in controlling time course of action and closer initial titration) as the second-line treatment for adults with ADHD. The Canadian ADHD Practice (CAP) Guidelines indicates amphetamine mixed salts (not available in SA), MPH OROS^®^ technology and atomoxetine as the first-line treatments, whereas other MPH preparations are considered second-line options.^72^

Currently, combinations of immediate and extended release preparations are often prescribed in adults – where the immediate release formulations are used as ‘top-up’ when the extended release formula is wearing off. The dose of stimulants should be individually adjusted, based on efficacy and tolerability – with a general recommended dose of 1 mg/kg.^73^ The range of half-lives of medications suggest that it is more practical to consider maximum dose in terms of the maximum taken at each time point, the length of effect of each dose on the control of ADHD symptoms and the number of doses required to provide sufficient control throughout the day – rather than a maximum based on mg/kg per day.^67^

#### Lisdexamphetamine (Vyvanse^®^)

Lisdexamphetamine is a prodrug metabolised by red blood cells to yield its active metabolite, d-amphetamine and L-lysine.^74^ A number of randomised, double-blind, placebo-controlled trials and open-able long-term studies have confirmed efficacy and tolerability for adults with ADHD,^75,76,77^ whereas a meta-analysis confirmed an effect size of 0.8.^70^ Furthermore, Jasinski and Krishnan^78^ documented a lower liability for recreational abuse – which would position this drug as one of the treatments of choice for patients with a dual diagnosis of ADHD and a substance abuse disorder. The NICE guidelines^37^ recommends dexamphetamine as the second-line treatment for adults who develop intolerable side effects to MPH. Common side effects of lisdexamphetamine include insomnia and irritability, whereas dizziness, appetite suppression with weight loss, headache and other gastrointestinal side effects have also been reported. There have been post-marketing reports of psychosis, aggression, depression, exacerbation of tics and cardiovascular side effects such as palpitations, hypertension and myocardial infarction with sudden death.

#### Atomoxetine (Strattera^®^)

For adults who do not respond to stimulant treatment or who have a condition in which a stimulant is contra-indicated, the non-stimulant atomoxetine is an appropriate alternative.^67^ The BAP guidelines^50^ and Maudsley guidelines^60^ recommend atomoxetine as the first-line treatment in patients with comorbid SUD and as the treatment of choice in patients with comorbid anxiety disorders, severe tics or when there is a risk of diversion of medication. The NICE guidelines^37^ as well as ENAA^67^ considers atomoxetine as a second-line agent for patients with insufficient response to, or those who cannot tolerate MPH.

In a meta-analysis of atomoxetine in the treatment of ADHD in children and adolescents, non-stimulants had a significant lower effect size than those of short-acting and long-acting stimulants (0.39, 0.96 and 0.73 respectively).^79^ However, in a meta-analysis comparing pharmacotherapeutic options in adults with ADHD, the effect size of atomoxetine was 0.59 versus an effect size of 0.67 for stimulants. However, in a recent systematic review,^80^ criticism against previous studies was raised. When the duration of studies was increased, and treatment naïve patients analysed separately, the effect size of atomoxetine improved to 0.9, which is very similar to the effect size of 1.0 of MPH. In SA, atomoxetine is still being underutilised^19^ – which may be a reflection of lack of knowledge and experience with regard to efficacy and effectiveness. It may also reflect patients’ preference for a medication which leads to immediate results – which stimulants provide.

Side effects of atomoxetine appear reflective of increased noradrenergic tone and include dry mouth, insomnia, nausea, decreased appetite, constipation, decreased libido, dizziness, sweating and rare cases of hepatotoxicity.^81^

#### Other agents

Although other drugs such as clonidine, guanfacine, TCAs, modafanil and venlafaxine are mentioned in international guidelines as the third-line treatment for ADHD,^50,67,72^ none of these are registered for the treatment of adult ADHD in SA and use will be off-label – at the discretion of the treating physician. Anticonvulsants, mood stabilisers, selective serotonin reuptake inhibitors and antipsychotic drugs have not been shown to be effective for primary symptoms of ADHD. They may, however, be useful in treating some comorbid conditions that patients with ADHD have.

α**_2_-noradrenergic agonists:** Clonidine (Dixarit®) has demonstrated efficacy in childhood ADHD, especially for cases marked by severe hyperactivity and aggression.^82^ However, there is an absence of literature on efficacy and tolerability in adults with ADHD.

Results from clinical trials have demonstrated efficacy for guanfacine (not available in SA) in combined (hyperactive-impulsive-inattentive) ADHD in children and adolescents, but it appears to be less effective in the inattentive subtype of ADHD. The response rate in children was 50% – 60%.^83,84^ In adults, the effect size was comparable to placebo.^85^ Potentially problematic side effects include hypotension, bradycardia, dizziness, somnolence, sedation, fatigue, dry mouth, nausea and abdominal pain. Guanfacine is the only ADHD medication which is associated with moderate weight gain.

**Tricyclic antidepressants:** Most studies of TCAs found a moderate to robust reduction in ADHD symptomatology in children with a moderate to strong treatment effect. The more selective noradrenergic drugs, that is, imipramine (Ethipramine®, Tofranil®) and the active metabolite desipramine, are more effective and better tolerated. A target dose of 200 mg/day is indicated. TCAs have negligible abuse liability and efficacy for comorbid anxiety and depression.^86^

**Modafanil (Provigil®):** Although efficacy for this wake-promoting drug in the treatment of ADHD in children has been demonstrated,^87^ studies in adults failed to demonstrate efficacy in reducing ADHD symptoms.^88,89^

**Selective serotonergic and noradrenergic reuptake inhibitors:** Venlafaxine (e.g. Effexor**®**, Venlor**®**) appears to be mildly efficacious in moderating ADHD symptom, with studies indicating a measurable reduction in symptoms at doses of 75 mg/day – 150 mg/day.^5,90,91^

**Noradrenergic dopaminergic reuptake inhibitors:** Bupropion hydrochloride (Wellbutrin**®**) has demonstrated moderate effect in the treatment of ADHD with a 42% reduction in ADHD rating, with 52% of subjects considered responders at a mean dose of 362 mg/day.^92,93,94,95^ Dosing appears to be optimal at 400 mg/day – 450 mg/day and there is a delay in onset of action. In a meta-analysis^96^ found the number needed to treat 4.6 with a discontinuation rate no higher than placebo. In a systematic search of studies, Peterson, McDonagh and Fu^71^ found the pooled effect size for bupropion to be 1.87. Side effects include insomnia, edginess and a low risk for seizures. Bupropion should be considered in adults with comorbid mood disorders (depression or bipolar disorder) or SUD.

**Monoamine oxidase inhibitors:** Some studies^97,98^ have suggested effectiveness of monoamine oxidase inhibitors (such as pargyline, deprenyl and selegiline) in the treatment of adult ADHD, but their potential to cause hypertensive crisis and the dietary restrictions pertaining to tyramine-containing foods, seriously limit their use.^99,100^

### Non-pharmacological interventions

#### Psychotherapy

Pharmacotherapy alone is not sufficient to stabilise the many problems of adults with ADHD, nor does current research support the efficacy of psychotherapeutic treatments as sole treatment for adult ADHD.^68^ The multimodal treatment study in ADHD^101^ has confirmed definitive evidence for the beneficial effect of psychotherapeutic interventions in childhood ADHD. However, evidence in adult ADHD is sparse. Various forms of structured, intensive and skills-based treatment may improve the likelihood of attaining remission, especially when combined with medication.^53^ The effects of therapy on the core symptoms of ADHD seem substantial, with potential effects on comorbid symptoms.

The NICE guidelines,^37^ ENAA^67^ and BAP guidelines^50^ recommend a multi-modal treatment approach with psychosocial treatments as complementary to psychopharmacological interventions to provide support, improve acceptance of diagnosis, and treat comorbidities and residual symptoms (which do not require, or do not tolerate, additional psychopharmacological treatments).

Although different approaches have been used, the majority of evidence is for structured CBT interventions, with modified CBT plus medication demonstrating greater benefits than psychotherapy or medication alone particularly on the core symptoms of ADHD, with lesser effect on anxiety and depression.^102,103^ CBT strategies are focused on improving the core symptoms of ADHD through self-instructional training and memory aids to improve attention, techniques to reduce impulsivity (e.g. ‘stop and think’), diaries and time schedules to improve organisational skills, and assertiveness and social skills training to improve communication skills. Group and individual CBT demonstrated similar outcomes.^104^

Mindfulness awareness therapy is a complementary intervention that not only improves affect and mood regulation in adults with ADHD, but also sustained attention and quality of life.^105^ There is also some evidence for the use of dialectical behaviour therapy,^106^ psychoeducation and organisational skills teaching^107^ and cognitive remediation programmes.^108^ Cognitive remediation provides techniques focussing on retraining cognitive function, teaching internal and external compensatory strategies, and restructuring the physical environment to maximise functioning. However, it is important to note that skills-based therapy and psychodynamic therapy^109^ may have a negative effect on self-esteem.

Formal studies of the effectiveness of coaching and psychoeducation have not been performed, but many adults with AHDD report that they gain benefit from these approached. Coaching is a structured, supportive therapy that can be offered individually or by group sessions. The purpose of coaching is to learn new problem-solving skills for identified practical problems.

It is optimal to pursue an individually tailored problem-oriented approach to psychosocial interventions. This may include individual, family, and group therapy, social skills training, occupational therapy and liaison with employees and academic institutions, and coaching.

#### Neurofeedback

Neurofeedback (NF) has been investigated as a possible alternative treatment for ADHD in children.^110^ NF is thought to reduce behavioural problems associated with the core symptoms of ADHD through enabling the patient to acquire skills to self-regulate certain brain activity patterns. In a meta-analysis, large effect sizes for impulsivity and inattention, and a medium effect size for hyperactivity were reported for theta and beta, sensorimotor rhythm and slow cortical potentials (SCP) protocols in ADHD.^111^ A recent study^112^ has evaluated the effectiveness of 30 sessions of SCP NF in 24 adults with ADHD. In this study, significant symptom reduction (self-rated and third-party rated) was observed with medium to large effect sizes after treatment and 6 months post treatment with regard to clinical symptoms, as well as reaction time, reaction time variability and contingent negative variation. Fourteen participants experienced a symptom reduction of over 25% and symptoms of six participants remitted, that is, they did not meet criteria for an ADHD diagnosis anymore. However, in a systematic review and meta-analyses of randomised controlled trials,^113^ NF did not demonstrate any statistically significant improvement on ADHD measures with blinded assessment in placebo- and non-placebo controlled trials.

#### Exercise

A recent meta-analysis investigating the effects of acute exercise on cognitive performance in healthy adults revealed that cognitive performance, particularly executive functioning tasks, was improved after 20 minutes of exercise.^114^ In humans and in animal models, exercise has been found to have wide-ranging cellular, structural, functional and cognitive benefits. These include enhanced frontal lobe volume, executive functioning, brain-derived neurotrophic factor (BDNF) levels, cerebral blood flow, and dopamine and other monoamine modulation.^115^

There is also growing body of research indicating positive effects of acute aerobic activity and longer-term activity on neuropsychological function in ADHD. Existing studies examining effects of physical activity on ADHD have generally studied children and adolescents, with some emerging work in adults with ADHD. Studies have shown that in children with ADHD, moderate-to-vigorous physical activity improves planning, and working memory, inhibitory control, novel problem-solving, processing speed and academic performance,^116,117,118,119^ whereas acute high intensity physical activity can improve sustained attention independent of stimulant treatment.^120^

In adults with ADHD, there is preliminary evidence that individuals who report more frequent physical activity experience reduced impulsivity, worry and intrusive thoughts,^18^ whereas acute moderate intensity exercise facilitated performance on executive functioning.^121,122^ Although specific recommendations regarding frequency, intensity or duration of exercise in ADHD have not been established,^123^ there is growing consensus that regular exercise engagement is a powerful nonpharmacological treatment for management of ADHD symptoms and promotion of cognitive health.

#### Complementary and alternative medications

In a systematic review and meta-analyses of randomised controlled trials of dietary and psychological interventions,^113^ 2904 published randomised controlled trials of dietary (restricted elimination diets, artificial food colouring exclusion and free fatty acid supplementation) and psychological interventions (attention and working memory training, cognitive training, NF and behavioural interventions) for ADHD were screened. Of these, 54 trials met inclusion criteria for the analysis. Two different analyses were performed: one where the outcome measure based on ADHD assessments was completed by parents or teachers, and the other where the best possible blinded assessments (i.e. placebo- and non-placebo controlled trials where the assessments were made by individual blinded to treatment) of improvement on ADHD measures were included. Although all dietary and psychological interventions produced statistically significant effects, in the latter group only free fatty acid supplementation and artificial food colouring exclusion remained significant. It therefore seems that free fatty acid supplementation produced small but significant reductions in ADHD symptoms even with probably blinded assessments, although the clinical effects of these effects remain to be determined. Artificial food colouring exclusion produced larger effects but often in individuals selected for food sensitivities.

N-3 omega-3 polyunsaturated fatty acids (n-3 PUFAs) are promoted as cognitive enhancers in the general population, as well as for patients with neurocognitive deficits such as ADHD. In a recent systematic review and meta-analysis,^124^ only marginal evidence was found for n-3 PUFA supplementation in those who are n-3 PUFA deficient. No significant benefit was found for the general population, nor those with ADHD.

Although many individuals, with and without ADHD, report benefits from ‘brainsmart food’ such as mentat, zinc, l-carnitine, vitamin B6, magnesium, ginkgo biloba, ginseng, passionflower, acetyl l-carnitine(ALCAR), dimethylethanolamine (DMAE), L-theanine, dosahexaenoic acid (DHA), citicoline, curcumin, phosphatidylserine, vincopetine, L-alpha-glycerylphosphorylcholine, bacopa monnieri and huperzine A, formal studies of the effectiveness of these agents are lacking. At present, there is no consistent evidence from randomised control trials for the use of any food supplements.^67^

### Algorithm

**FIGURE 1:**
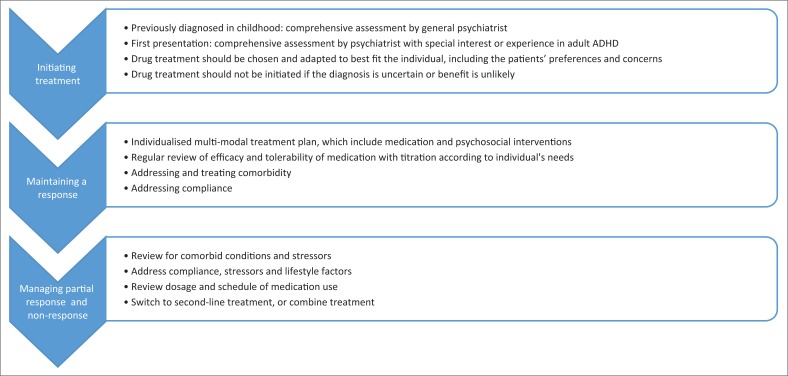
Management process.

See Figure 1 and Table 2.

**TABLE 2:** Medication schedule.

Substance	Trade name	Formulation	Dosing strategy
Methylphenidate	Ritalin^®^ IR HCL Douglas-methylphenidate^®^	Immediate release. Available in 10 mg tablets	Approximately 1 mg/kg/dose. Initiate at 5 mg bd or tds with daily or weekly increments according to efficacy and tolerability (max 100 mg/day po)
	Ritalin^®^ LA	Extended release. Available in 10 mg, 20 mg, 30 mg and 40 mg capsules	Once or twice daily dose at equivalent of total daily dose of IR
	Concerta^®^	Osmotic release system. Available in 18 mg, 27 mg, 36 mg and 54 mg capsules.	Once or twice daily dose at equivalent of total daily dose of IR.
Atomoxetine	Strattera^®^	Available in 10 mg, 18 mg, 25 mg, 40 mg, 60 mg and 80 mg capsules	Initiate at approximately 0.5 mg/kg/day in patients <70 kg with recommended daily dose 1.2 mg/kg/day. In patients >70 kg initiate at 40 mg/day with monthly increments of 20 mg/day to a maximum of 100 mg/day (maintain at least for 12 weeks before judging clinical response)
Lisdexamphetamine	Vyvanse^®^ *not available in South Africa	Available in 10 mg, 20 mg, 30 mg, 40 mg, 50 mg, 60 mg and 70 mg capsules	Initiate at 30 mg/day po. May be adjusted in increments of 10 mg or 20 mg at approximately weekly intervals up to maximum dose of 70 mg/day. In patients with severe renal impairment, the maximum dose should not exceed 50 mg/day po.
Bupropion	Wellbutrin^®^	Available in 150 mg slow release and 150 mg and 300 mg extended release tablets	Initiate at 150 mg/day po. Dosage may be adjusted in increments of 150 mg approximately monthly intervals up to maximum dose of 300 mg/day.
Venlafaxine	Effexor^®^ Venlor^®^	Available in 75 mg and 150 mg extended release capsules	Initiate at 75 mg/day po. Dosage may be adjusted in increments of 75 mg at approximately monthly intervals up to maximum dose of 450 mg/day.
Imipramine/Desipramine	Tofranil^®^, Ethipramine^®^	Available in 10 mg and 25 mg tablets	Initiate at 20 mg to 70 mg/day (10 mg in elderly patients) and increase gradually to a maintenance dose of 100 mg to 150 mg/day po (50 mg in elderly patients)
Clonidine	Dixarit^®^	Available in 0.025 mg tablets	Initiate at 0.025 mg bd po and increase gradually to a maximum dose of 0.075 mg bd po.
Modafinil	Provigil^®^	Available in 100 mg tablets	Initiate at 100 mg/day po. Can be increased to 200 mg/day po.

### Special populations

#### Comorbidity

The order of treatment of ADHD and comorbid disorders depends on the severity of the different disorders and clinical judgement on which disorder is driving the current level of behavioural impairments or mental state changes. The presence of comorbidity increases the likelihood of the need for combination therapy – both in terms of psychopharmacological agents and psychotherapeutic approaches. In initiating medication for comorbid conditions, an individualised approach with regard to effectiveness, risk-benefit profile and tolerance should be considered.

In the presence of comorbid disorders, a risk assessment should be done. Severe mood disorders, severe anxiety disorders (with panic attacks) and psychotic disorders should be treated prior to treating ADHD. Milder disorders (e.g. mild depression and anxiety) may be deferred until after treatment of ADHD as treating the ADHD might alleviate other symptoms.^67^ A sequential approach to initiating medication is recommended. In the case of psychotic disorders or severe bipolar disorders, stimulants should be used with caution.

Patients with ADHD and comorbid SUD often present with more severe SUD characterised by early onset, extended duration of the SUD, greater impairment and shorter transition from substance use to dependence. In cases of comorbid SUD, the SUD can be treated either before initiating treatment for ADHD, or simultaneously. Close supervision is advised when treating ADHD patients with SUD with stimulants and in cases where diversion or abuse is a particular concern, atomoxetine may be selected as the first-line treatment. The concerns of some professionals that use of stimulants in ADHD may lead to drug abuse either by sensitisation or as a gateway to other drugs is not supported by evidence. Long-acting stimulants with a lower abuse potential, lisdexamphetamine and bupropion are preferred over short-acting stimulants. Concomitant psychosocial interventions are crucial.

#### Pregnancy and lactation

All the stimulants, atomoxetine, bupropion, modafinil and other pharmacological agents discussed in these guidelines (see section Pharmacological treatment) are considered ‘category C’ by the FDA, that is, animal studies have reported some harm, without having any robust evidence in human studies.^125^ Although continuation of treatment during pregnancy may therefore pose a risk to the child, discontinuation may increase harmful behaviours related to the mother’s mental state.^50^ It is therefore important to consider the risk–benefit profile of not treating the mother, for example, poor risk management, dangerous driving, illicit drug use, alcohol and tobacco use, increased levels of distress and self-injurious behaviour. Non-pharmacological strategies should be explored, and liaison with the obstetrician is important.

## Summary

Adult ADHD is a chronic, costly and debilitating disorder – if untreated. Recognition of adult ADHD as a chronic disorder which needs chronic treatment is crucial.

A comprehensive diagnostic assessment and diagnostic certainty prior to initiating drug treatment are crucial. This is not possible during the average 15-min general practitioner consultation, and it is therefore strongly advised that the diagnosis of adult ADHD and treatment initiation should be made by a psychiatrist well versed in the complexities of ADHD and the comorbidity thereof. Raising the diagnostic bar for adult ADHD will also prevent the scripting of medication for patients who use the medication for reasons other than the treatment of ADHD (e.g. cognitive enhancement) which artificially escalates the prevalence and costs of ADHD.

Once a proper treatment plan has been established, and a patient is contained within a multi-disciplinary and multi-modal (psychosocial intervention and an optimal medication regime) basket of care, follow-up can take place on primary healthcare level – leading to further cost savings to schemes and reducing pressure on resources (manpower and financial) at specialist level.^19^ Ongoing treatment (which includes compliance to treatment) is crucial in preventing complications and long-term costs.
